# Effects of host sex, body mass and infection by avian *Plasmodium* on the biting rate of two mosquito species with different feeding preferences

**DOI:** 10.1186/s13071-019-3342-x

**Published:** 2019-03-12

**Authors:** Rafael Gutiérrez-López, Josué Martínez-de la Puente, Laura Gangoso, Ramón Soriguer, Jordi Figuerola

**Affiliations:** 1 Department of Wetland Ecology, Estación Biológica de Doñana (EBD-CSIC), C/Américo Vespucio 26, 41092 Seville, Spain; 2 Present Address: Theoretical and Computational Ecology, Institute for Biodiversity and Ecosystem Dynamics, University of Amsterdam, Science Park 904, 1098 XH Amsterdam, The Netherlands; 3 Department of Ethology & Biodiversity Conservation, Estación Biológica de Doñana (EBD-CSIC), C/Américo Vespucio 26, 41092 Seville, Spain; 4 CIBER de Epidemiología y Salud Publica, Seville, Spain

**Keywords:** *Aedes*, Avian malaria, *Culex pipiens*, Haemosporidians, Mosquito feeding patterns, *Ochlerotatus caspius*, Wild birds

## Abstract

**Background:**

The transmission of mosquito-borne pathogens is strongly influenced by the contact rates between mosquitoes and susceptible hosts. The biting rates of mosquitoes depend on different factors including the mosquito species and host-related traits (i.e. odour, heat and behaviour). However, host characteristics potentially affecting intraspecific differences in the biting rate of mosquitoes are poorly known. Here, we assessed the impact of three host-related traits on the biting rate of two mosquito species with different feeding preferences: the ornithophilic *Culex pipiens* and the mammophilic *Ochlerotatus* (*Aedes*) *caspius*. Seventy-two jackdaws *Corvus monedula* and 101 house sparrows *Passer domesticus* were individually exposed to mosquito bites to test the effect of host sex, body mass and infection status by the avian malaria parasite *Plasmodium* on biting rates.

**Results:**

*Ochlerotatus caspius* showed significantly higher biting rates than *Cx. pipiens* on jackdaws, but non-significant differences were found on house sparrows. In addition, more *Oc. caspius* fed on female than on male jackdaws, while no differences were found for *Cx. pipiens*. The biting rate of mosquitoes on house sparrows increased through the year. The bird infection status and body mass of both avian hosts were not related to the biting rate of both mosquito species.

**Conclusions:**

Host sex was the only host-related trait potentially affecting the biting rate of mosquitoes, although its effect may differ between mosquito and host species.

## Background

The blood-feeding behaviour of mosquitoes is a complex phenomenon that involves different steps. The initial seeking and location of hosts depends on the integration of chemical (e.g. CO_2_, odours) and visual cues (e.g. host size and plumage/pelage coloration) emitted by the host [1, 2]. In close proximity between mosquitoes and their hosts, odour, heat and host defensive behaviour may affect the final host choice and blood-feeding success of mosquitoes [3].

Under natural conditions, mosquitoes show different innate feeding preferences, with some species feeding mostly on mammals (mammophilic species, and some of them can be characterized as anthropophilic), while others preferring to bite birds (ornithophilic species), or even amphibians or reptiles, yet other species show a more opportunistic behaviour [4–8]. In addition to this broad tendency for particular host classes, mosquitoes bite certain host species at higher rates than those expected from their abundance [9–11]. For instance, Kilpatrick et al. [9] showed that American robins (*Turdus migratorius*) were more intensely bitten by *Culex pipiens* mosquitoes than European starlings (*Sturnus vulgaris*) in North America. Similarly, in Europe, the feeding preference of *Cx. pipiens* for blackbirds (*Turdus merula*) was higher than for European starlings [12]. Within host species, some individuals may receive most mosquito bites and, as a result, they may play a role as superspreaders when infected with vector-borne pathogens [13].

This heterogeneity in vector attraction and host use by mosquitoes could have important impacts on the dynamics of transmission of parasites causing human and animal diseases [14]. Among others, these factors may determine the transmission success of parasites such as protozoans (e.g. *Plasmodium* spp.) and filarial worms (e.g. *Dirofilaria* spp.) [2, 15].

Different non-mutually exclusive mechanisms may determine that an individual host receives more mosquito bites, such as, for instance, the use of habitats with higher abundance of mosquitoes, a higher emission of attractive cues, or a less intense or effective anti-mosquito behaviour than other conspecifics [7]. In addition, larger hosts (i.e. with a larger body mass, as a correlate of body size), may receive more bites by mosquitoes [16] probably due to the higher amounts of cues (e.g. CO_2_) released by larger individuals [17]. Different studies at the interspecific level have reported a positive relationship between host body mass and the feeding rate of different blood-sucking insects [18, 19]. However, very few studies have experimentally tested the relationship between species variation in body mass and the feeding rate of mosquitoes [20]. In addition, sex-specific morphological, physiological and/or behavioural characteristics could produce differences in the attraction of insect vectors [21]. These differences in vector attraction between host sexes have been argued as a potential explanation for the usually higher prevalence of blood parasites found in male than in female birds [22–24]. However, to the best of our knowledge, only Burkett-Cadena et al. [25] evaluated the effect of bird sex on the variation in mosquito biting preferences. By analysing the blood-meal origin of mosquitoes, authors found that blood meals were biased towards male birds, but only in mammophilic mosquitoes. However, the reasons of these differences remain unclear. Patterns found by Burkett-Cadena et al. [25] could be the result of a differential susceptibility, attraction and/or exposure of bird sexes to mosquito attacks or simply an unbalanced bird sex-ratio in the areas where mosquitoes were captured. Finally, the host infection status by vector-borne parasites may also influence the mosquito biting patterns, potentially determining the pathogen transmission success [26, 27]. For example, humans infected with *Plasmodium vivax* were more attractive to mosquito vectors [28]. However, studies with avian *Plasmodium* are less conclusive, because *Cx. pipiens*, the main vector of avian *Plasmodium*, was reported to preferentially bite chronically infected birds over uninfected individuals according to Cornet et al. [26, 27], but other studies have reported the opposite pattern [29] or even the absence of significant differences between infected and uninfected birds [30].

In this study, we experimentally assessed the impact of three host-related traits (body mass, sex and infection status by avian *Plasmodium*) on mosquito feeding patterns, while removing host anti-mosquito behaviour. We performed this study using two mosquito species with different feeding preferences: the ornitophilic *Cx. pipiens* and the mammophilic *Ochlerotatus* (*Aedes*) *caspius* [8, 31, 32]. We used two bird species as host models, the jackdaw (*Corvus monedula*) and the house sparrow (*Passer domesticus*). Both bird species are common hosts of avian malaria parasites [33, 34]. Based on previous evidence [19, 25–27], we predicted (i) a higher biting rate on birds in the ornithophilic *Cx. pipiens* than in the mammophilic *Oc. caspius*; (ii) a higher mosquito biting rate on heavier individuals; (iii) a higher biting rate on male birds over females, especially for *Oc. caspius*; and (iv) a higher biting rate on *Plasmodium-*infected birds than on uninfected individuals.

## Methods

### Mosquito collection and rearing

*Culex pipiens* and *Oc. caspius* larvae were collected from March to August in 2014 and 2016 in the natural reserve ‘La Cañada de los Pájaros’ (36°57′N, 6°14′W; Seville Province, Spain) and in marshlands of Huelva Province (37°17′N, 6°53′W), respectively. Larvae were transferred to the laboratory and kept in plastic trays with fresh or brackish water, respectively, and fed *ad libitum* with Mikrozell 20 ml/22 g (Dohse Aquaristik GmbH & Co. KG, Gelsdorf, Germany). Larvae and adult mosquitoes were maintained under standard conditions (28 ± 1 °C, 65–70% RH and 12:12 light:dark photocycle). Adult mosquitoes were anesthetized with ether and their sex and species identified based on morphology, on chilled Petri dishes using a stereomicroscope (Nikon SMZ645, Tokyo, Japan) following Schaffner et al. [35]. After identification, adult females were placed in insect cages (BugDorm-43030F, 32.5 × 32.5 × 32.5 cm; MegaView Science Co, Taichung, Taiwan) and fed *ad libitum* on 1% sugar solution. Twenty-four hours prior to each experiment, female mosquitoes were deprived from sugar solution. Laboratory maintained colonies of mosquitoes were not used to minimize the effects of artificial selection of mosquitoes with particular biting preferences [36, 37].

### Bird sampling and experimental procedure

The jackdaw is a non-migratory passerine bird, resident in Europe, western Asia and North Africa. It is 34–39 cm long and has a body mass of 181–257 g. This species is not sexually dimorphic. The house sparrow is also a non-migratory passerine, native to most Europe. It is 14–18 cm long and has a body mass of 21–31 g. Although body mass does not differ between sexes, adults of this species present strong sexual dimorphism in plumage coloration [38].

The jackdaws were caught from March to July 2014 in ‘La Cañada de los Pájaros’ using a walk-in trap, while the house sparrows were caught using mist nets from April to August 2014 in the same location, and from June to August 2016 in different localities from the Huelva Province. Birds were individually ringed with numbered metal rings weighed and blood sampled from the jugular vein using sterile syringes. The volume of blood obtained differed between species due to differences in body mass (1 ml in jackdaws and 0.2 ml in house sparrows). Female birds with brood patches were released immediately after capture and were not included in this study to reduce any impact on their reproductive performance. The experimental feeding trials were undertaken from 7:30 to 12:00 h (GMT + 1 h).

Individual birds were enclosed for 30 min in an insect cage (BugDorm-43030F, 32.5 × 32.5 × 32.5 cm, MegaView Science Co, Taichung, Taiwan) containing 53 ± 33.7 (mean ± SD) (range 1–152) mosquito females of either *Cx. pipiens* or *Oc. caspius*. The experimental feeding trials were developed in an environment with low light and no noise that could alter their behaviour. A number of previous studies have reported the ability of mosquito species including *Cx. pipiens* to feed on birds maintained in cages [39, 40]. Each bird was immobilized to prevent defensive behaviours against mosquitoes. Jackdaws were immobilized using non-permanent masking tape, with the wings attached to the body, the beak closed and legs held together. Un-feathered areas of the body (i.e. legs and eyes) remained uncovered during the trials, thus allowing mosquitoes to feed on the birds. House sparrows were immobilized using a cylinder made with 1 × 1 cm mesh, allowing mosquitoes to bite through. After the trials, birds were released at the same location of capture without any apparent sign of damage. Mosquitoes with a recent blood meal in their abdomen, including those showing partial and full engorgement, were counted and categorised as blood-fed mosquitoes.

### Molecular analyses

Genomic DNA was extracted from bird blood samples using Maxwell® 16 LEV Blood DNA Kit (Promega, Madison, WI, USA) [41]. Birds were molecularly sexed following Griffiths et al. [42]. The *Plasmodium* infection status of birds was assessed by the amplification of a 478 bp fragment of the mitochondrial cytochrome *b* gene following Hellgren et al. [43]. The presence of amplicons was verified in 1.8% agarose gels and positive samples were sequenced using BigDye technology (Applied Biosystems, Foster City, CA, USA) or the Macrogen sequencing service (Macrogen Inc., Amsterdam, Netherlands). Sequences were edited using the software Sequencher™ v.4.9 (Gene Codes Corp., Ann Arbor, MI, USA) and assigned to parasite genus after comparison with the GenBank database (National Centre for Biotechnology Information). Only birds infected with avian *Plasmodium* were included in this study, while birds infected or co-infected with *Haemoproteus* or *Leucocytozoon* were removed from the analysis.

We characterized the occurrence of *Cx. pipiens* biotypes in the area (i.e. La Cañada de los Pájaros) where larvae were collected in the context of other study. We used 140 mosquitoes captured and amplified the 5’ flanking region of the CQ11 microsatellite following [44, 45]. We found that *Cx. p. pipiens*, *Cx. p. molestus* and their hybrids were present in the area (frequency of 45, 10.7 and 44.3%, respectively). The biotypes of mosquitoes included in this study where not analysed due to the large sample size.

### Statistical analysis

The proportion of mosquitoes that bit house sparrows and jackdaws were compared separately for the two-mosquito species using Chi-square tests. We used generalized mixed linear models (GLMMs) with binomial error and logit link function to assess the effect of mosquito species and bird characteristics on mosquito biting rates. Analyses were performed in R software v.3.2.5 [46] with the package *lme4* [47]. First, we compared the biting rates of the two mosquito species on birds. Models included the mosquito biting rate as the dependent variable, expressed as the number of mosquitoes that bit on the focal bird with respect to the number of mosquitoes that did not bite this individual using the *cbind* function. Due to the differences in the method used to immobilize each bird species and their clear differences in body size, separated models were fitted for jackdaws and house sparrows. In each case, bird body mass and the date (day 1 = 1st January) on which each trial was conducted were included as covariates and bird sex, *Plasmodium* infection status (infected/uninfected) and mosquito species (*Cx. pipiens*/*Oc. caspius*) were included as fixed factors. Because house sparrows were captured in two different years, the variable year was included as a fixed factor in the model assessing the biting rate of mosquitoes exposed to this bird species. We also included the two-way interactions between mosquito species and host sex and between mosquito species and infection status in the models. Bird identity was included as a random term to correct for the overdispersion shown when using both binomial and quasibinomial distributions (dispersion parameter > 7.21) [48]. The variables body mass and date were scaled for each species by the standard deviation and mean-centred to normalize the variable distribution. The jackdaw population studied here was subjected to a medication experiment with birds either injected immediately before exposure to mosquitoes with a sub-curative dose of primaquine or treated as controls. This treatment did not affect the mosquito biting rate (*Z* = -1.2, *Estimate* = -0.62, *P* = 0.26), thus this factor was not included in further analyses. The potential effect of the different *Plasmodium* lineages on the mosquito biting rate was not analysed due to the low sample size available for the different combinations between bird and mosquito species (see Table 1).

**Table 1:** Number of individuals infected with each *Plasmodium* lineage in this study

*Plasmodium* lineages	Jackdaws		House sparrows	
	*Cx. pipiens*	*Oc. caspius*	*Cx. pipiens*	*Oc. caspius*
SGS1	21	5	17	13
GRW11			7	2
COLL1			6	2
PADOM01			3	2
DELURB5	2	1		
PADOM02			3	
GRW4		1		
Total infected	30		55	

## Results

Seventy-two jackdaws (34 males and 38 females) and 101 house sparrows (66 males and 35 females) were included in this study. Of these, 30 jackdaws (41.7%) and 55 house sparrows (54.5%) were infected with avian *Plasmodium*. Overall, 7 *Plasmodium* lineages were detected (Table 1). A total of 9153 mosquito females were exposed to the birds, including 6387 *Cx. pipiens* and 2766 *Oc. caspius*. Of these, 630 (9.9%) *Cx. pipiens* and 633 (22.9%) *Oc. caspius* fed on blood (Table 2), including 294 (46.7%) *Cx. pipiens* and 436 (68.9%) *Oc. caspius* which fed on jackdaws and 336 (53.3%) *Cx. pipiens* and 197 (31.1%) *Oc. caspius* which fed on house sparrows (Table 2).

**Table 2:** Summary data of mosquitoes biting jackdaws and house sparrows used in this study with respect to host sex and infection status by avian *Plasmodium* parasites

			*n*	No. of mosquitoes in each assay per box (mean ± SE)	No. of engorged mosquitoes per box (mean ± SE)
Jackdaws					
*Cx. pipiens*	Sex	Male	26	59.1 ± 6.5	7.0 ± 2.6
		Female	29	49.7 ± 6.2	3.6 ± 1.0
	Infectious status	Uninfected	32	58.7 ± 5.9	4.9 ± 2.9
		Infected	23	47.8 ± 7.1	6.0 ± 3.5
*Oc. caspius*	Sex	Male	8	62.9 ± 12.0	17.0 ± 4.8
		Female	9	58.1 ± 11.2	33.5 ± 6.2
	Infectious status	Uninfected	10	71.9 ± 10.7	21.3 ± 5.8
		Infected	7	45.9 ± 12.8	30.3 ± 7.3
House sparrows					
*Cx. pipiens*	Sex	Male	41	57.3 ± 5.4	4.9 ± 0.9
		Female	20	53.0 ± 7.7	6.9 ± 1.3
	Infectious status	Uninfected	25	52.0 ± 6.8	4.6 ± 1.2
		Infected	36	58.6 ± 5.8	6.2 ± 1.0
*Oc. caspius*	Sex	Male	25	44.4 ± 6.8	4.2 ± 1.2
		Female	15	41.7 ± 9.4	6.1 ± 1.6
	Infectious status	Uninfected	21	32.7 ± 7.5	3.6 ± 1.3
		Infected	19	55.2 ± 8.2	6.4 ± 1.4

**Fig. 1:**
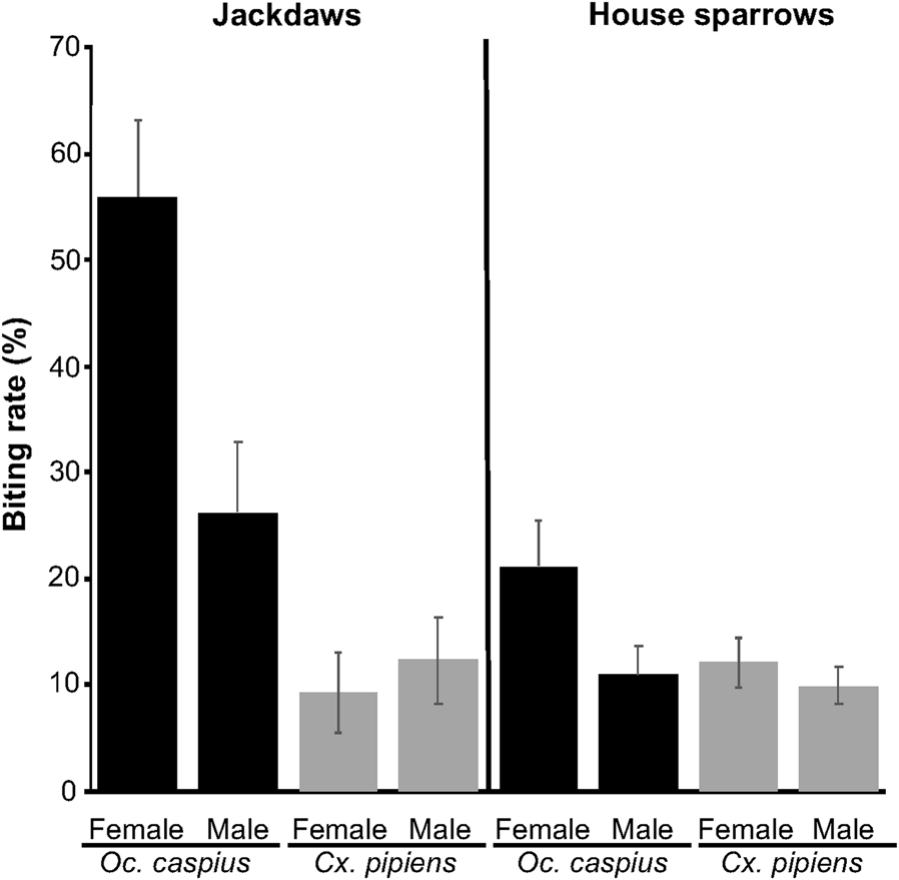
Biting rates of *Ochlerotatus caspius* and *Culex pipiens* mosquitoes on female and male jackdaws and house sparrows

The biting rate of *Oc. caspius* on jackdaws was higher than on house sparrows (*χ*^2^ = 15.43, *df* = 1, *P* < 0.001), while no differences were found for *Cx. pipiens* (*χ*^2^ = 0.04, *df* = 1, *P* = 0.84; Fig. 1). The mammophilic *Oc. caspius* showed a significantly higher biting rate than the ornithophilic *Cx. pipiens* (*Z* = 4.22, *Estimate* = 1.00, *P* < 0.001; Fig. 1).

The effects of bird traits on mosquito biting rates were studied separately for each bird species because of the differential methodology (i.e. immobilization) used in each species and the differences reported above. In the case of jackdaws, *Oc. caspius* showed a significantly higher biting rate than *Cx. pipiens* (Table 3). In addition, *Oc. caspius* showed a higher biting rate on female than on male jackdaws, while non-statistically significant differences were found for *Cx. pipiens* (Table 3, Fig. 1). Host infection status by avian *Plasmodium*, body mass and date were not significantly related to mosquito biting rates (Table 3). For house sparrows, we found no differences in the biting rate between *Cx. pipiens* and *Oc. caspius*. Date was the only variable significantly related to mosquito biting rate, with an increase in biting rates as the seasons progressed. Host sex, body mass and infection status by avian *Plasmodium* were not significantly related to mosquito biting rates on house sparrows (Table 3, Fig. 1).

**Table 3:** Results of GLMMs analysing mosquito biting rates in relation to mosquito species (*Ochlerotatus caspius* and *Culex pipiens*) and birds’ body mass, sex, *Plasmodium* infection status, date on which each trial was conducted, and year of bird capture (for jackdaws, year was not included because all the individuals were captured the same year). The interactions between variables are indicated with *. Significant effects are highlighted in bold

Explanatory variables	Jackdaws				House sparrows			
	Estimate	SE	*Z-*value	*P-*value	Estimate	SE	*Z-*value	*P-*value
Mosquito species	2.51	0.56	4.50	**< 0.001**	0.51	0.54	0.94	0.35
Body mass	0.75	0.82	0.92	0.36	1.46	0.80	1.84	0.07
Sex	0.04	0.39	0.10	0.92	-0.57	0.36	-1.59	0.11
Infection status	0.03	0.36	0.07	0.95	0.02	0.37	0.05	0.96
Date trial	0.86	0.82	1.04	0.30	2.11	0.71	2.96	**0.003**
Year	–	–	–	–	0.25	0.36	0.71	0.48
Mosquito species*sex	-1.37	0.69	-1.99	**0.047**	0.28	0.56	0.60	0.62
Mosquito species*infection status	0.83	0.70	1.19	0.23	-0.44	0.57	-0.77	0.44
Explained variance (*R*^2^)	0.20				0.07			

*Abbreviation*: SE, standard error

## Discussion

Identifying the potential causes underlying the non-random biting patterns of mosquitoes is essential to understand the dynamics of transmission of avian *Plasmodium* and other vector-borne pathogens [13]. Here, we tested how three important avian traits (i.e. body mass, sex and the infection status by *Plasmodium*) in two bird species affect the biting rates of two mosquito species potentially involved in the transmission of avian malaria parasites [32]. Nonetheless, other factors not quantified in this study, such as the size of the blood meal, could also be important in the parasite transmission success and should be therefore considered in future studies.

The biting rate of the mammophilic *Oc. caspius* on jackdaws was higher than the biting rate of the ornithophilic *Cx. pipiens*, while non-significant differences were found when mosquitoes faced house sparrows. Although most of the blood meals of *Oc. caspius* analysed in different studies was derived from mammals, birds including chickens and house sparrows represented between 9.1–19.9% of the blood meals in this species [6, 49]. This pattern clearly contrasts with that of *Cx. pipiens*, for which birds represent between 85.1–91.67% of the blood meals [6]. In this study, the *Cx. pipiens* biotypes were not considered although potential differences in the feeding preferences have been reported [50]. However, mosquitoes in this study were captured in an area where the two biotypes (*Cx. pipiens pipiens* and *Cx. pipiens molestus*) and their hybrids coexist. A previous study conducted in southern Spain did not find significant differences in the proportion of blood meals from birds between *Culex pipiens* biotypes [45]. Our results clearly support the ability of *Oc. caspius* to feed on birds, at least when they are not allowed to choose between other host classes (i.e. mammals). This fact is especially relevant as our study focuses on the biting rate of mosquitoes and not on the feeding preferences of these species. Contrary to *Cx. pipiens*, *Oc. caspius* is traditionally considered as an aggressive mosquito and an important nuisance to human populations [51], but experimental studies supporting this assumption are scarce. Differences in the biting rate between mosquito species could be associated with their life history traits and breeding requirements, especially those related to the availability of water sources. While *Oc. caspius* depends on tidal cycles and uses temporal flooded areas for larval development [52], *Cx. pipiens* uses more permanent water sources [53] and, consequently, their life-cycle may be less time-constrained. In addition, it is possible that a differential activity pattern between mosquito species could have affected our results, with *Oc. caspius* showing a strong peak of activity during the day while *Cx. pipiens* peaks its activity at night and sunset [49]. Although this possibility could potentially explain the differences found between species, the biting rates of *Cx. pipiens* found in this study are similar to those found in a previous experiment developed during the night [30]. The variation in climatic conditions throughout the year is expected to affect the phenology of both the host and the vector and thus may potentially influence the host-vector interactions. We found that mosquito biting rates increased over time (i.e. from spring to autumn), but only when exposed to house sparrows. In this regard, Edman [54] reported differences in the feeding patterns of *Culex nigripalpus* depending on the season. In our case, this effect seems to be associated to particular host-mosquito assemblages, since it was apparent in only one of the two bird species studied. Nevertheless, it is important to highlight that the relevance of this variable should be considered low, as the model including the significant effect of the date explained only 7% of the variance.

The fact that differences in biting patterns were only detected when mosquitoes faced jackdaws, the larger host species, suggests these may be related to differences in the amounts of cues emitted by each bird species. In close proximity to their hosts, the relevance of visual and thermo-sensory stimulation of mosquitoes increases with respect to larger distances. Moreover, the use of multiple sensory cues may increase the likelihood of mosquito feeding success [3]. Due to their larger size, jackdaws may emit a higher amount of attractants, including CO_2_, heat and odours, than house sparrows, potentially leading to the differences found here.

Previous studies have reported a positive relationship between host body mass and biting rates of blood-sucking insects [19, 55], although this pattern usually corresponds to studies comparing different host species. As expected from its larger size, jackdaws were bitten at a higher rate than house sparrows by *Oc. caspius*, although we did not find significant differences for *Cx. pipiens.* The amount of bare skin exposed, usually positively correlated with body mass, may affect mosquito feeding success [56]. In fact, body mass was a key variable explaining the prevalence of infection by the mosquito-borne pathogen West Nile virus in birds [57]. Further support for this possibility can be derived from the study by Yan et al. [58], who found that *Cx. pipiens* fed more often on birds with longer tarsus, suggesting that larger areas of exposed skin are important for determining biting patterns. In this respect, Burkett-Cadena et al. [39] found that the proportion of bites on nestlings with respect to the adult female in the nest increased as they grow, suggesting that size in addition to the exposed skin may be key factors influencing mosquito feeding patterns. However, we did not find any significant relationship between the biting rate of mosquitoes and bird body mass at the intraspecific level. In this regard, Lalubin et al. [29] found that the attraction of *Cx. pipiens* to house sparrows was not significantly associated with their body mass. This suggests that, at short distances, the slight intraspecific differences in body mass are probably less important than other cues determining mosquito bites, like heat, humidity or odour [3].

As predicted, host sex influenced the biting rates of *Oc. caspius* mosquitoes when facing jackdaws. However, and in contrast to our prediction, this mosquito species preferred to bite females than males, but these differences were not found when mosquitoes were exposed to house sparrows. The biting rates of *Cx. pipiens* were not significantly related to host sex. Burkett-Cadena et al. [25] found male-biased blood meals in mosquitoes (64% of the blood meals analysed derived from male birds), although they suggest this could be due to skewed sex ratios in wild birds. However, there are not significant sex-biased differences in the feeding patterns of bird-biting mosquitoes, including *Culex* species [25]. Moreover, Simpson et al. [20] concluded that bird sex has no effect on the probability of *Cx. pipiens* choosing an individual over its partner. The preference of mammophilic mosquitoes for a particular sex of bird could be associated with the sexual differences in the composition of odour profiles. Among other factors, the volatile and non-volatile substances of the secretions of the preen gland may affect the feeding preferences of blood-sucking insects [59, 60] and their composition differ between bird sexes [61, 62]. Differences in the response of *Oc. caspius* and *Cx. pipiens* to secretions of the preen gland could explain, at least in part, discrepancies found between mosquito species [63].

We did not find support for a relationship between avian *Plasmodium* infection status and mosquito biting rates. In view of previously reported results, it is unclear whether avian malaria infection enhances [26, 27] or decreases [29] the mosquito attraction towards the infected host. Thus, the host manipulation hypothesis pointing to an increase in *Plasmodium* transmission success through a higher attractiveness of infected hosts to mosquito bites remains an open question [64, 65]. Different authors have used diverse experimental procedures to test for the effect of avian infections on mosquito attraction; some of these are summarized in Table 4. For example, some authors have used dual-port olfactometers [29] while others have allowed mosquitoes to bite birds exposed under different situations (e.g. immobilized birds) [26, 27], with all these differences potentially affecting the conclusions obtained. In addition, different host-vector assemblages have been used, including different species of wild and domestic birds and insect vectors [26, 27, 29, 30, 66, 67]. Moreover, the results of our study should be interpreted with caution, as the host individuals used in our experiments were naturally infected with different lineages of *Plasmodium* and probably were in different stages of infection, which could potentially affect host attractiveness for vectors. Due to the low prevalence of some *Plasmodium* lineages in birds, we were unable to analyse possible differences in the effects of the different *Plasmodium* lineages on mosquito feeding preference. However, as far we know, no previous study has found differential bird attractiveness for vectors depending on the avian *Plasmodium* linage infecting them. In addition to changes in the amount and quality of cues emitted by infected hosts, differences in the intensity of defensive behaviour associated with the infection status might explain differences in their susceptibility to mosquito attacks [68, 69]. For example, Day et al. [68] found that malaria-infected mice were more lethargic and less likely to defend against mosquitoes. In our study, birds did not show symptoms of lethargy and all were immobilized to prevent anti-mosquito defensive behaviour. Therefore, the possibility of changes in host defensive behaviour owing to *Plasmodium* infection was ruled out in this study. Additionally, it is possible that the potential differences in the emission of cues between infected and uninfected hosts [70] can only be appreciated by mosquitoes at large distances, when host-seeking behaviour is mainly based on olfactory clues [3] or may be only evidenced when performing dual-choice experiments [26], which is not the case for our study.

**Table 4:** Studies on the effects of avian malaria and malaria-like infections in birds on the biting patterns of different insect vectors. Studies reporting positive, negative or absence of associations between parasite infections and the susceptibility of birds to insect attacks are reported

Effects	Vectors	Bird species	Parasite species	Methods	Results	Reference
Positive	*Cx. pipiens*	Canaries	*P. relictum* SGS1	Biting rate on immobilized birds exposed in pairs (blood-meal identification)	Infected birds were more bitten by uninfected mosquitoes than uninfected birds^a^	[26]
	*Cx. pipiens*	Canaries	*P. relictum* SGS1	Biting rate on immobilized birds exposed in pairs (blood-meal identification)	Infected birds were more bitten by infected and uninfected mosquitoes	[27]
	*Cx. pipiens*	House sparrows	*Plasmodium*	Biting rate on birds exposed in pairs (blood-meal identification)	The intensity of infection but not the parasite prevalence determined the mosquito biting rates^b^	[30]
Negative	*Cx. pipiens*	Great tits	*Plasmodium*	Attractivity of birds exposed in pairs (olfactometer)	Uninfected birds attracted more mosquitoes than infected ones	[29]
	Biting midges; blackflies	Blue tits	*Haemoproteus majoris*	Insect abundance in nest boxes of wild birds	A higher abundance of *Culicoides* was found in nests of primaquine-medicated females than controls; this was not the case for blackflies	[66]
	Biting midges	Blue tits	*Haemoproteus*	Insect abundance in nest boxes of wild birds	A higher abundance of *Culicoides festivipennis* was found in nests from primaquine-medicated birds than controls^c^	[67]
No effect	*Cx. pipiens*; *Oc. caspius*	House sparrows; jackdaws	*Plasmodium*	Biting rate on immobilized birds exposed individually	No effect of the infection status on biting rate	This study

^a^Significant results were only found when mosquitoes were exposed to birds with chronic infections

^b^Significant differences were only found when mosquitoes were exposed to infected birds treated with the antimalarial drug primaquine and control birds

^c^Effects were only found for *C. festivipennis*, but not for the other species included in the study

## Conclusions

This study highlights that the magnitude and direction of the effects of host traits such as body mass, sex or the infection status by the mosquito-borne avian *Plasmodium* on the feeding patterns of mosquitoes are far from being generalizable. Only sex was associated to differences in mosquito biting rates, and this effect was only detected for one of the mosquito species studied here. Consequently, the biting patterns of mosquitoes may differ according to vector and host species characteristics. The reasons underlying the preference of mammophilic mosquitoes for individuals of a particular sex are unclear and need detailed analyses with regard, for instance, to the olfactory cues released by male and female birds.
